# Objective assessment of cognitive fatigue: a bibliometric analysis

**DOI:** 10.3389/fnins.2024.1479793

**Published:** 2024-11-01

**Authors:** Jia-Cheng Han, Ke Bai, Chi Zhang, Na Liu, Guan Yang, Yu-Xuan Shang, Jia-Jie Song, Dan Su, Yan Hao, Xiu-Long Feng, Si-Rui Li, Wen Wang

**Affiliations:** Department of Radiology, Functional and Molecular Imaging Key Lab of Shaanxi Province, The Second Affiliated Hospital of Air Force Medical University, Xi’an, Shaanxi, China

**Keywords:** cognitive fatigue, mental fatigue, assessment, detection, bibliometric analysis, citation analysis

## Abstract

**Aim:**

The objective of this study was to gain insight into the nature of cognitive fatigue and to identify future trends of objective assessment techniques in this field.

**Methods:**

One thousand and eighty-five articles were retrieved from the Web of Science Core Collection database. R version 4.3.1, VOSviewer 1.6.20, CiteSpace 6.2.R4, and Microsoft Excel 2019 were used to perform the analysis.

**Results:**

A total of 704 institutes from 56 countries participated in the relevant research, while the People’s Republic of China contributed 126 articles and was the leading country. The most productive institute was the University of Gothenburg. Johansson Birgitta from the University of Gothenburg has posted the most articles (*n* = 13). The PLOS ONE published most papers (*n* = 38). The Neurosciences covered the most citations (*n* = 1,094). A total of 3,116 keywords were extracted and those with high frequency were mental fatigue, performance, quality-of-life, etc. Keywords mapping analysis indicated that cognitive fatigue caused by continuous work and traumatic brain injury, as well as its rehabilitation, have become the current research trend. The most co-cited literature was published in Sports Medicine. The strongest citation burst was related to electroencephalogram (EEG) event-related potential and spectral power analysis.

**Conclusion:**

Publication information of related literature on the objective assessment of cognitive fatigue from 2007 to 2024 was summarized, including country and institute of origin, authors, and published journal, offering the current hotspots and novel directions in this field.

## 1 Introduction

Cognitive fatigue is mainly caused by the accumulation of metabolites in the brain after prolonged mental activity or excessive stress. It is limited solely to a mental state arising from a behavioral situation that includes a long-term, continuous, and repetitive performance of some mental tasks (Jalali et al., 2023; Pan et al., 2021; Wang and Pei, 2014). Prolonged cognitive fatigue can lead to decreased sleep duration, lower sleep quality, and impaired cognitive function, leading to decreased mental and physical performance in different settings (Lorist et al., 2005). In extreme cases, cognitive fatigue gives rise to catastrophic events such as traffic accidents or medical errors (Mabry et al., 2022; Pyper and Paterson, 2016).

Considering this fact, plenty of research on cognitive fatigue detection has been steadily rising, especially the objective analysis such as fatigue events measures, eye-tracking measures, self-assessment of cognitive fatigue, and noninvasive EEG measures. In recent years, with a significant improvement in computational performance and feature extraction methods, the number of varied features extracted from the objective signal of cognitive fatigue state is still increasing (Stancin et al., 2021). Cognitive fatigue detection based on objective indicators with a high temporal resolution shows a more promising future than other methods. However, there still lacks the whole figure about the objective assessment of cognitive fatigue.

Bibliometrics is the quantitative analysis of academic publications which was first defined in 1969 and gained widespread acknowledgment by academia (Raisig, 1962). As scientific research in the objective assessment of cognitive fatigue is proliferating, bibliometric analysis of this field may provide direction for research questions and raise awareness of research trends. Therefore, it is necessary to conduct a bibliometric analysis on the objective assessment of cognitive fatigue (Chen et al., 2022a, b). To our knowledge, no bibliometric analysis focusing on this topic has been published till now. To fill this gap, this bibliometric analysis constructed a global map of the scientific publications on the objective assessment of cognitive fatigue. The research status, hotspots, and future research trends in this field were expected to be determined through the analysis.

## 2 Methods

### 2.1 Data source and literature inclusion criteria

**Figure 1:**
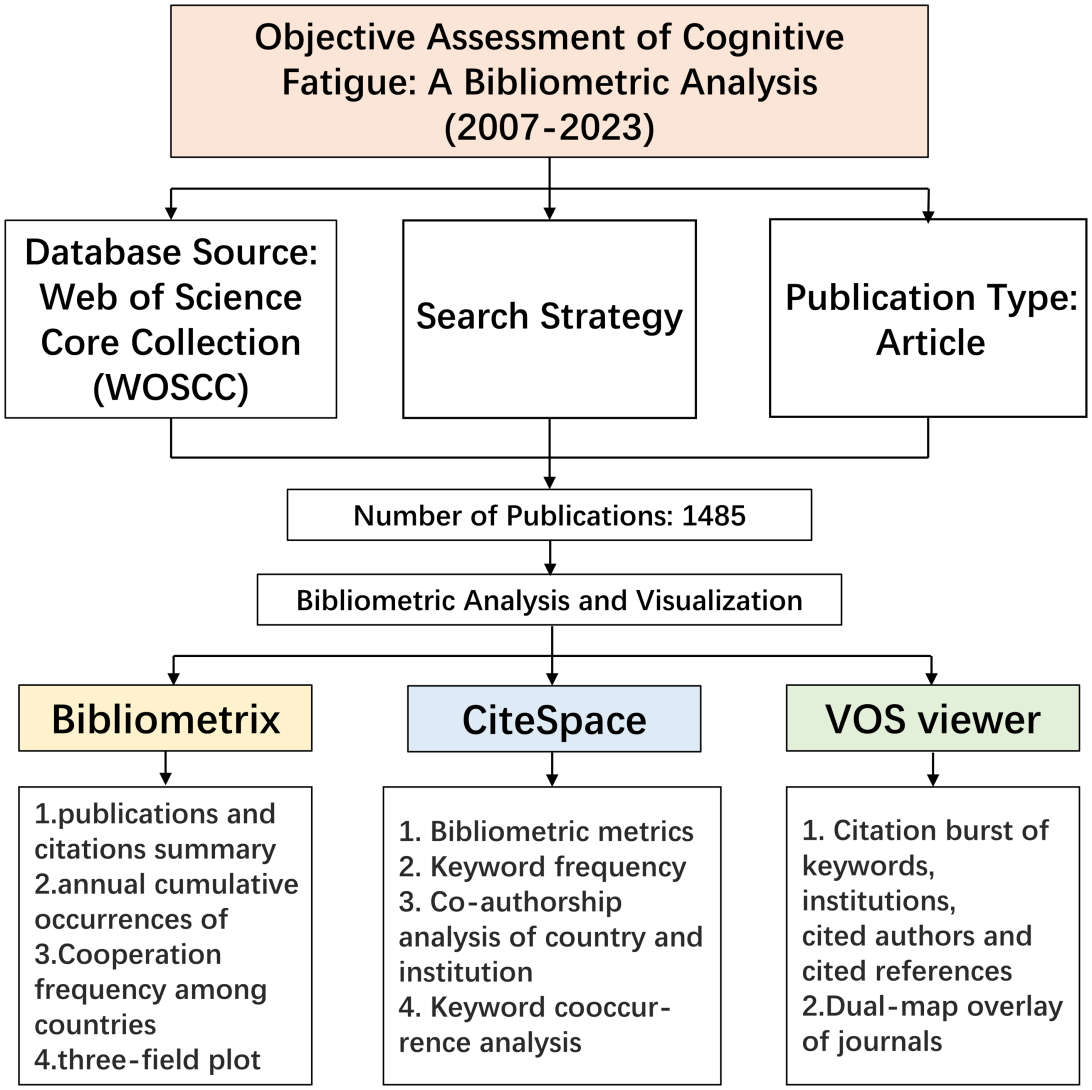
Flow-chart of the study.

As shown in Figure 1, we retrieved the data from the Web of Sciences Core Collection (WOSCC) database from its inception to November 26, 2023. WOSCC is one of the most widely used databases in academics, which provides leading journals and detailed information about worldwide publications (Xu J. G. et al., 2022). WOSCC was chosen as the primary database for this study because of its comprehensive coverage of multiple academic journals and its frequent use by researchers. In addition, WOSCC provides the most comprehensive and reliable bibliometric analysis data related to objective assessment of cognitive fatigue compared to other databases such as Scopus, Medline, and PubMed, including data on co-cited references and co-cited journals (Yeung, 2019). We searched the WOSCC to identify all studies on the objective assessment of cognitive fatigue. Since most researchers considered that “cognitive fatigue,” “mental fatigue,” and “brain fatigue” are conceptually similar, union logic was applied to the 3 keywords by inserting the conjunction “OR” (Van Cutsem et al., 2017; Marcora et al., 2009; Brownsberger et al., 2013; Pageaux et al., 2015; Smith et al., 2015; Bijleveld, 2023; Sengupta et al., 2017; Sievertsen et al., 2016). Likewise, 15 synonyms screened from research papers were linked by the logic word “OR,” which have similar meaning to the word “assessment.” In order to establish the relationship between cognitive fatigue and objective assessment, intersection logic was applied to the two groups of keywords by the logic word “AND.” The search strategy was: (TS = (“cognitive fatigue”) OR TS = (“mental fatigue”) OR TS = (“brain fatigue”)) AND (TS = (detect) OR TS = (detection) OR TS = (monitor) OR TS = (recognize) OR TS = (recognition) OR TS = (identification) OR TS = (identify) OR TS = (evaluation) OR TS = (evaluate) OR TS = (assess) OR TS = (assessment) OR TS = (track) OR TS = (tracking) OR TS = (estimation) OR TS = (estimate)). No time limit was implemented, while the language of literature was limited to English.

The retrieved articles from the databases were exported to EndNote X9 [Thomson Reuters (Scientific) LLC Philadelphia, PA, United States] for further categorization. Two independent reviewers (HJC and BK) reviewed the literature for relevant articles by reading the titles, abstracts, and full texts if necessary, so that all literature on the objective assessment of cognitive fatigue was included as much as possible. Discrepancies in the process were resolved by discussion between the two authors until they reached an agreement, and a list is output through EndNote X9. Publication types were limited to articles only, which means reviews, meetings, abstracts, editorial materials, letters, news reports, and book reviews were excluded.

### 2.2 Data collection and statistical analysis

The final list was exported to Microsoft Excel 2019 (Microsoft, Redmond, WA, United States, Microsoft. com) spreadsheets. The following data from each publication were extracted from the list: authors, titles, sources of journals, keywords, impact factors (IF) of journals, citations, references, institutes of authors, country of authors, co-citation authors, and co-citation journals, etc. The overall design of this study referred to several previous bibliometric research (Derviş, 2020; Chen et al., 2022a; Shukla et al., 2020; Jin et al., 2022). The IF of journals was obtained from the 2023 Journal Citation Reports.

### 2.3 Visualization and network mapping

Bibliometrix is an R package containing a series of functions for scientometric quantitative research. It was used to summarize the number of publications, citations of bibliometric analysis, annual cumulative occurrences, the top keywords/terms, and the cooperation frequency among countries in this study. In addition, it was also used to visualize a three-field plot of the Keywords Plus analysis (Derviş, 2020).

VOSviewer is a visual tool used to make science mapping analyses of publications in the journal because it has a robust user graphic interface and mapping visual capability (van Eck and Waltman, 2010). VOSviewer (1.6.20) was used to extract critical information, specifically for high-frequency fields such as countries, institutes, and keywords (Shukla et al., 2020). VOSviewer graphically represents them through bibliometric analysis in an easy-to-understand manner. We make a network map for visual analysis and further analyze the potential clusters to identify a research field trend and measure the proximity degree. The interpretation of a visual network map is based on four characteristics: size, colors, distance, and connecting line thickness. A node represents a specific term, such as country, author, or keyword. Before being imported into VOSviewer to generate maps, all data were normalized. We standardized different expressions about the same author or keyword into a uniform expression to reduce the bias in data analysis, which was completed artificially by the authors (Goo et al., 2021).

CiteSpace software was developed by Dr. Chen, a scholar at Drexel University, United States. The software uses Java to conduct visual analyses of scientific references. CiteSpace is one of the most sought-after bibliometric tools for investigating topic evolution, and it is usually used to detect bursts (Wang Z. Q. et al., 2021). CiteSpace 6.2.R4 was used in this study to detect bursts for co-occurrence items, such as authors, institutes, keywords, and co-cited references. Bursts are defined as characteristics that are cited frequently over a period of time. Dual-map overlay of journals was also created by CiteSpace (Jin et al., 2022).

## 3 Results

### 3.1 Annual publications

A total of 1,485 original articles were retrieved from the WOSCC database, which were published from 7 May 2007 to 14 November 2023 with the H-index of 1,086. Among the documents included, the earliest record about the objective assessment of cognitive fatigue was published in 2007 (Bailey et al., 2007). Of the 1,485 articles, 78 (5.3%) were published between 2007 and 2009, 857 (57.7%) were published between 2010 and 2020, and 550 (37%) were published between 2021 and 2023. The year with the highest annual number of publications was 2022.

**Figure 2:**
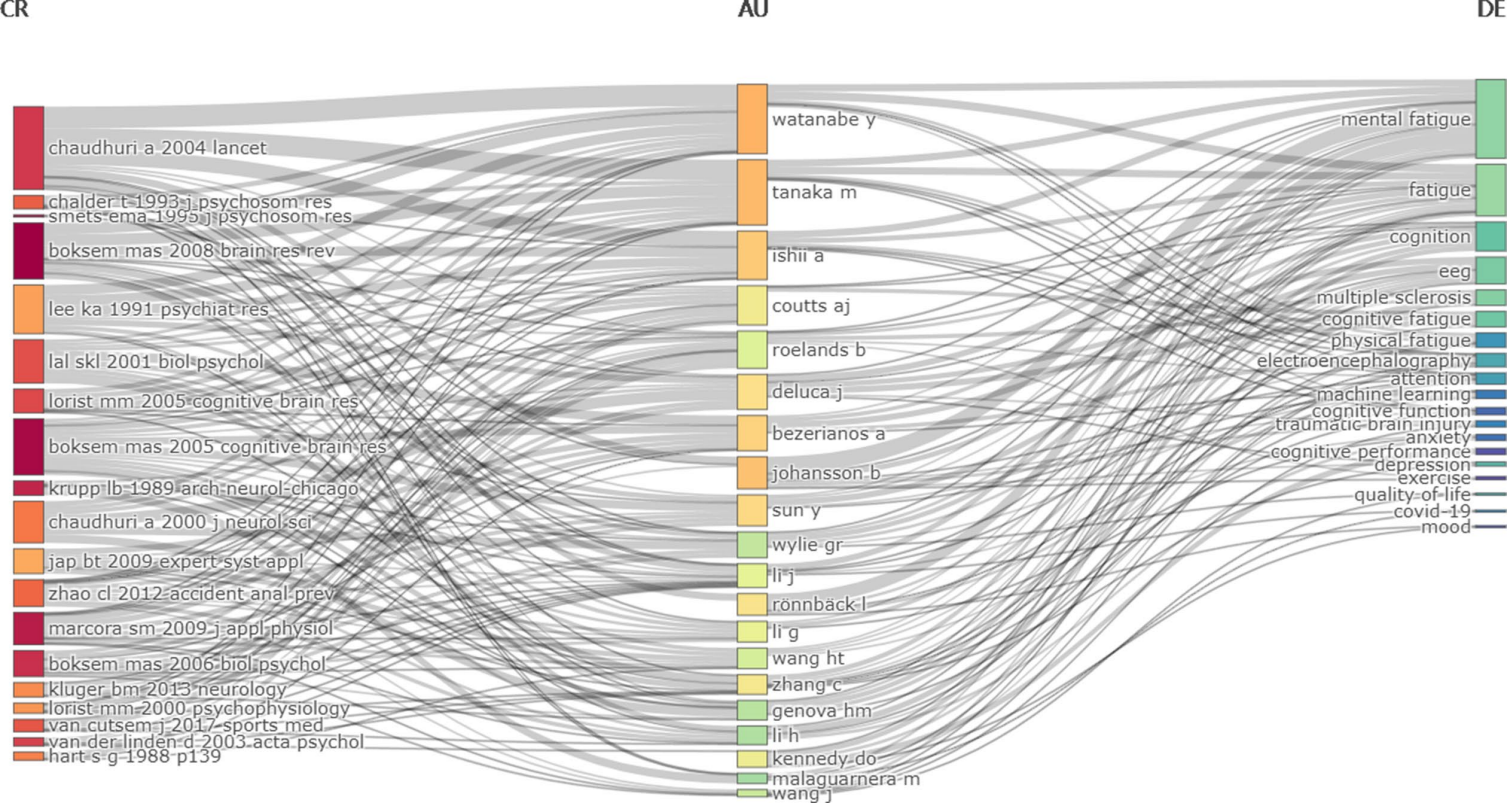
Number of publications per year and the cumulative amount.

Figure 2 shows the growth of annual publications on the objective assessment of cognitive fatigue, with a steady increase in the cumulative number of publications. There was a significant increase in publications between 2021 and 2022, indicating that research on cognitive fatigue assessment has increased in the past 3 years. A curve is fitted between time and annual publications using an exponential function to understand cognitive fatigue assessment research trends better. The equation presented in Figure 2 predicts the annual publications with an R-square of 96.4%. Table 1 shows the frequency and centrality of the top 10 topic categories related to cognitive fatigue assessment, mainly in the fields of neurosciences (*n* = 134), psychology (*n* = 61), clinical neurology (*n* = 56) and sport sciences (*n* = 50). In addition, MF rehabilitation is mainly applied in neurosciences (Centrality = 0.39).

**Table 1:** Top 10 main categories according to the number of publications.

Rank	Category	n	Centrality
1	Neurosciences	134	0.39
2	Psychology	61	0.12
3	Clinical Neurology	56	0.15
4	Sport Sciences	50	0.03
5	Engineering, Electrical, and Electronic	38	0.22
6	Computer Science, Artificial Intelligence	34	0.33
7	Behavioral Sciences	31	0.05
8	Rehabilitation	30	0.11
9	Engineering, Biomedical	29	0.21
10	Multidisciplinary Sciences	23	0.15

### 3.2 Countries/institutes’ collaboration

**Figure 3:**
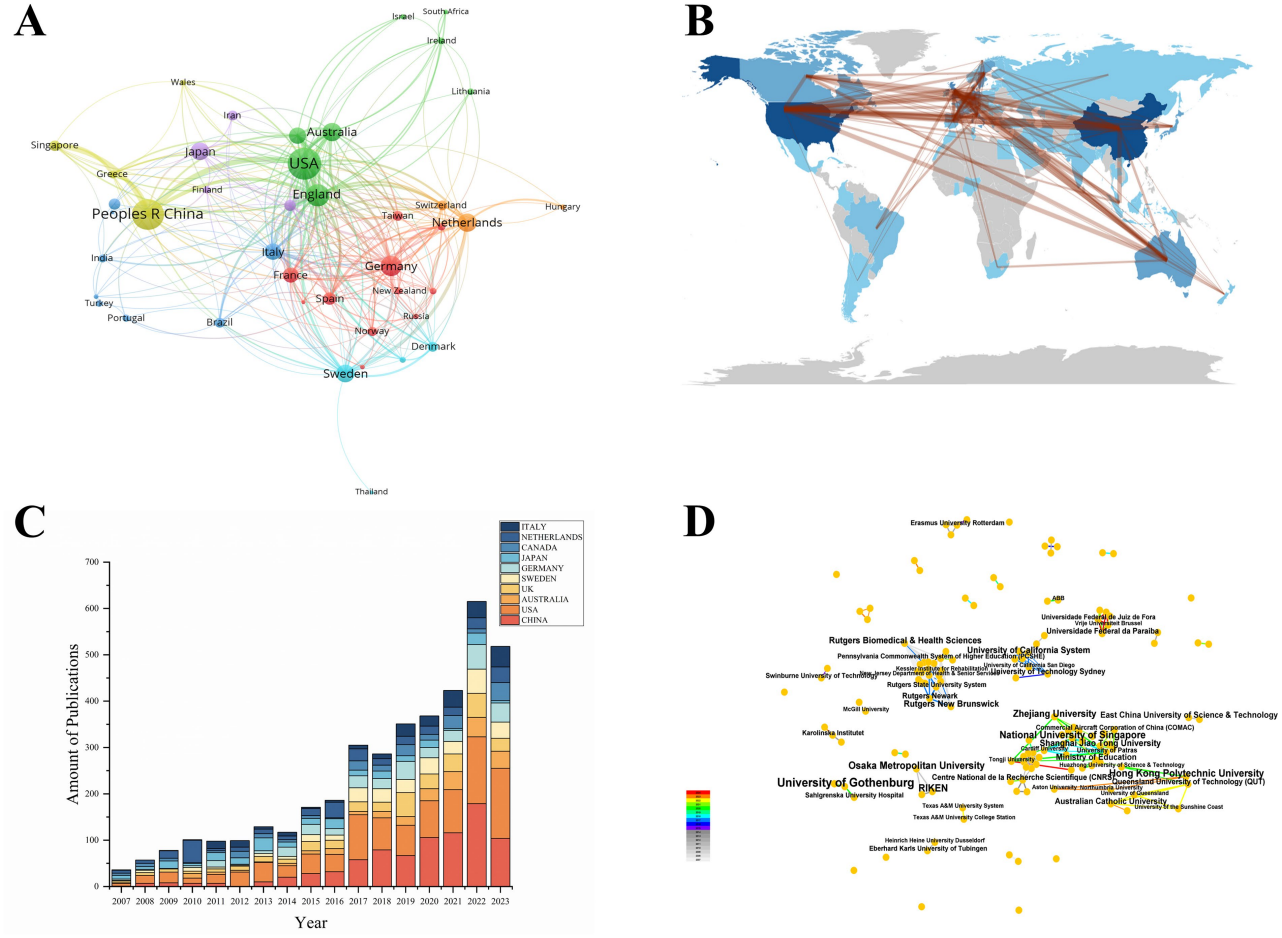
(A) Network map of cooperation between countries. (B) Countries’ collaboration map. (C) The top 10 contributing countries to publications over time. (D) The cooperation network visualization map of institutions.

According to the search results, there are a total of 704 institutes from 56 countries. We have listed the top 10 countries and institutes in terms of the publication number (Table 2; Table 3). The People’s Republic of China (PRC) has the largest number of publications, contributing 126 articles. And the United States of America (USA) ranks second (*n* = 87), followed by Australia (*n* = 42). The University of Gothenburg published the largest number of articles, which had published 17 papers in this field, followed by RIKEN, the National University of Singapore, Osaka Metropolitan University, and Hong Kong Polytechnic University (*n* = 10). International collaboration was analyzed based on country or institute using VOSviewer and CiteSpace. Figure 3A presents a visualization map of collaboration between different countries. According to the countries’ collaboration, the USA, the PRC, England, Germany, Australia, Netherlands, Japan, Sweden, and Italy maintained intimate cooperation with other countries. The thickness of connecting lines represents the strength of collaboration between the nodes. The analysis found that England had the strongest collaboration network, represented by a total link strength of 183, followed by the USA (total link strength of 135) and the PRC (total link strength of 101). Figure 3B presents a global perspective of publications in this field, lines between countries indicate cooperation, and line color and width indicate frequency division. This figure indicates a wide range of cooperation among countries around the world in this field, especially between the USA and PRC. Figure 3C shows the top 10 contributing countries to publications over time, indicating that publications from PRC in the field have increased significantly in recent years, and other countries have also increased in annual publications.

**Table 2:** The top 10 contributing countries to publications.

Rank	Country	n	Citations
1	CHINA	126	4,405
2	USA	87	6,146
3	AUSTRALIA	42	1,340
4	ENGLAND	41	2,515
5	SWEDEN	36	1,485
6	GERMANY	34	1800
7	JAPAN	33	1,492
8	CANADA	24	1,197
9	NETHERLANDS	24	1,542
10	ITALY	23	1,185

**Table 3:** The top 10 contributing institutes and countries to publications.

Rank	Institutes	Country	n	Citations
1	University of Gothenburg	Sweden	17	874
2	RIKEN	Japan	10	595
3	National University of Singapore	Singapore	10	939
4	Osaka Metropolitan University	Japan	10	639
5	Hong Kong Polytechnic University	CHINA	10	327
6	Zhejiang University	CHINA	9	254
7	Shanghai Jiao Tong University	CHINA	7	411
8	Rutgers New Brunswick	USA	7	755
9	Xi’an Jiaotong University	CHINA	7	353
10	University of California System	California	7	332

According to the analysis of the number of papers issued by 704 institutes, 438 (62.22%) institutes only participated in publishing one study, 184 (26.14%) institutes published two-three studies, 25 (3.55%) institutes published four studies, and there were 56 (7.95%) institutes published more than four studies. The National University of Singapore and Cardiff University started early cooperation and had close ties with each other since then. On the other hand, Chinese researchers at Zhejiang University, Shanghai Jiao Tong University, and Tongji University conducted more recent research in this field and had close ties with each other (Figure 3D). The modularity score was significant (*Q* = 0.8652) and the silhouette score was significant (*S* = 0.9853).

### 3.3 Authorship collaboration

**Figure 4:**
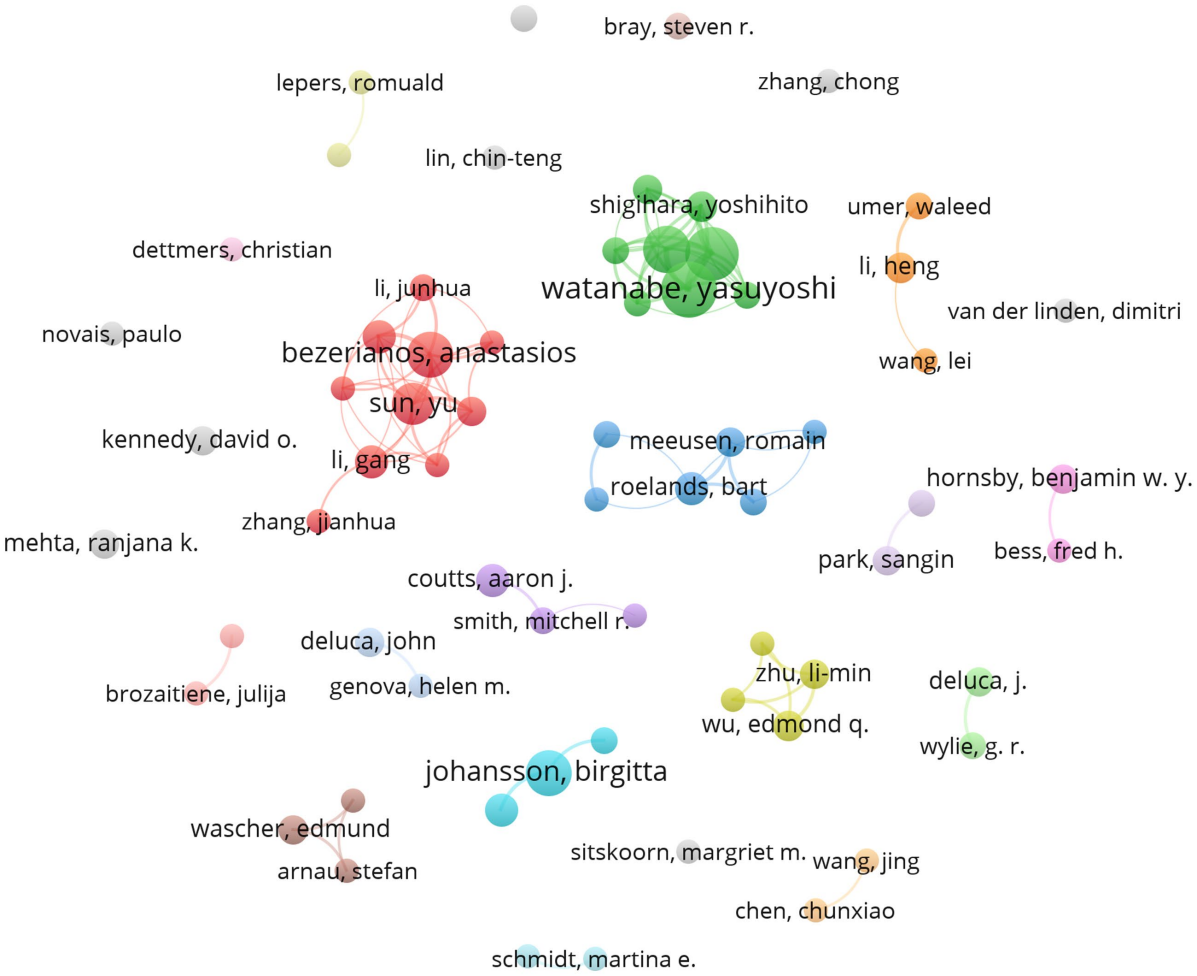
Network map of cooperation between authors.

A total of 2,141 authors participated in the research field, and 1846 (86.22%) authors with a minimum of one study. In addition, 203 authors published two studies, 41 authors published three studies, 25 authors published four studies, and 24 authors published five to thirteen studies during the investigating period. Johansson Birgitta ranked first among all authors and could be considered as the most productive, who published 13 studies with a total of 676 citations, followed by Tanaka Masaaki (documents = 10, citations = 604), Watanabe Yasuyoshi (documents = 10, citations = 647) and Ishii Akira (documents = 10, citations = 395) (Table 4). Figure 4 shows the network cooperation among 66 authors with a minimum number of five documents (including five). A total of 16 cooperative author groups were detected, and the top 10 authors were from five clusters, of which five were from the same cluster. The author’s collaboration network visualization maps can provide information on potential collaborators, showing the dominant collaboration between small groups while lacking large-scale collaboration.

**Table 4:** The top 10 contributing authors and their institutes to publications.

Rank	Author	Institutes	n	Citations
1	Johansson Birgitta	University of Gothenburg	13	676
2	Tanaka Masaaki	Osaka Metropolitan University	10	604
3	Watanabe Yasuyoshi	Osaka Metropolitan University	10	647
4	Ishii Akira	Osaka Metropolitan University	10	395
5	Ronnback Lars	University of Gothenburg	9	602
6	Li Heng	Huazhong University of Science and Technology	8	189
7	Bezerianos Anastasios	Zhejiang University	8	507
8	Sun Yu	Zhejiang University	8	361
9	Wu Edmond Q	Shanghai Jiao Tong University	7	212
10	Coutts Aaron J	University of Technology Sydney	7	573

### 3.4 Tables journals and co-cited journals

The retrieved data was published in 606 different journals. Among them, 360 (59.41%) journals published only one article, 174 (28.71%) journals published two to four studies, and 72 (11.88%) journals published more than five (including five) studies. Table 5 lists the top 10 journals and co-cited journals. PLOS ONE published the largest number of papers (38 publications), followed by the International Journal of Environmental Research and Public Health (31 publications). The top 10 most productive journals published 223 papers in the field. The average IF with the top 10 journals was 3.8402. In the citation analysis, 137 journals with a total number of citations of more than 100 (including 100). The top five most cited journals were Neuroimage (*n* = 1,094), PLOS ONE (*n* = 923), Journal of Psychosomatic Research (*n* = 701), Psychophysiology (*n* = 681), and Biological Psychology (*n* = 629). Neuroimage is in a leading position in the number of citations. Among the top 10 journals, six are from the USA, two are from the UK, one is from the Netherlands, and one is from Ireland.

**Table 5:** The top 10 journals and co-cited journals.

Rank	Journal	n	Citations	Country	IF	Co-cited journal	Citations	Country	IF
1	PLoS ONE	38	923	USA	3.7	Neuroimage	1,094	USA	5.7
2	International Journal of Environmental Research and Public Health	31	191	Switzerland	1.802	PLOS ONE	923	USA	3.7
3	Frontiers in Human Neuroscience	25	401	Switzerland	2.9	Journal of Psychosomatic Research	701	UK	4.7
4	Multiple Sclerosis and Related Disorders	24	106	UK	4.0	Psychophysiology	681	USA	3.7
5	Scientific Reports	24	2	UK	4.6	Biological Psychology	629	NETHERLANDS	2.6
6	Multiple Sclerosis Journal	18	596	UK	5.8	Multiple Sclerosis Journal	596	UK	5.8
7	Brain Injury	17	395	UK	1.9	Neurology	539	USA	9.9
8	Nutrients	17	119	Switzerland	5.9	Medicine & Science in Sports & Exercise	518	USA	4.1
9	Sensors	15	2	Switzerland	3.9	Sleep	460	USA	5.6
10	Ieee Access	14	123	USA	3.9	Clinical Neurophysiology	431	IRELAND	4.7

**Figure 5:**
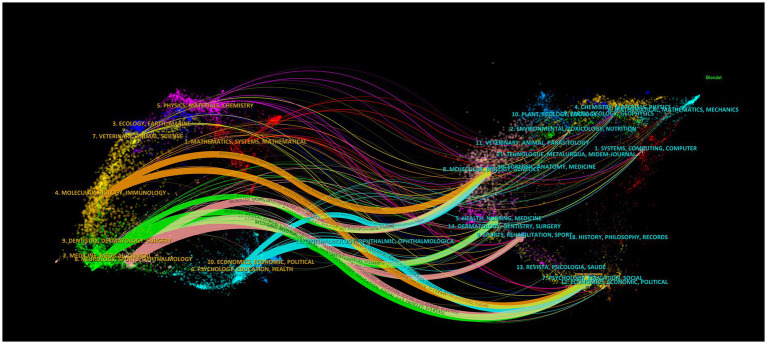
The dual-map overlay of journals.

To analyze the distribution of published and cited journals more specifically, we carried out dual-map overlaying using the visual analysis method (Figure 5). The map on the left represents the citing journal, while the map on the right represents the cited journal. The label represented the subject covered by the journal. Colored curves represent paths of references, originating from the citing map on the left and pointing to the cited map on the right. There were 10 main citation paths in the current map. The path referred to these papers published in molecular, biology, immunology, medicine, medical, clinical, neurology, sports and ophthalmology. And the most cited papers were mainly published in molecular, biology, genetics, health, nursing, medicine, sports, rehabilitation, psychology, education and social. This is the development mode of the integration of multiple disciplines into a discipline, but also a reflection of interdisciplinary.

### 3.5 Keywords mapping

**Figure 6:**
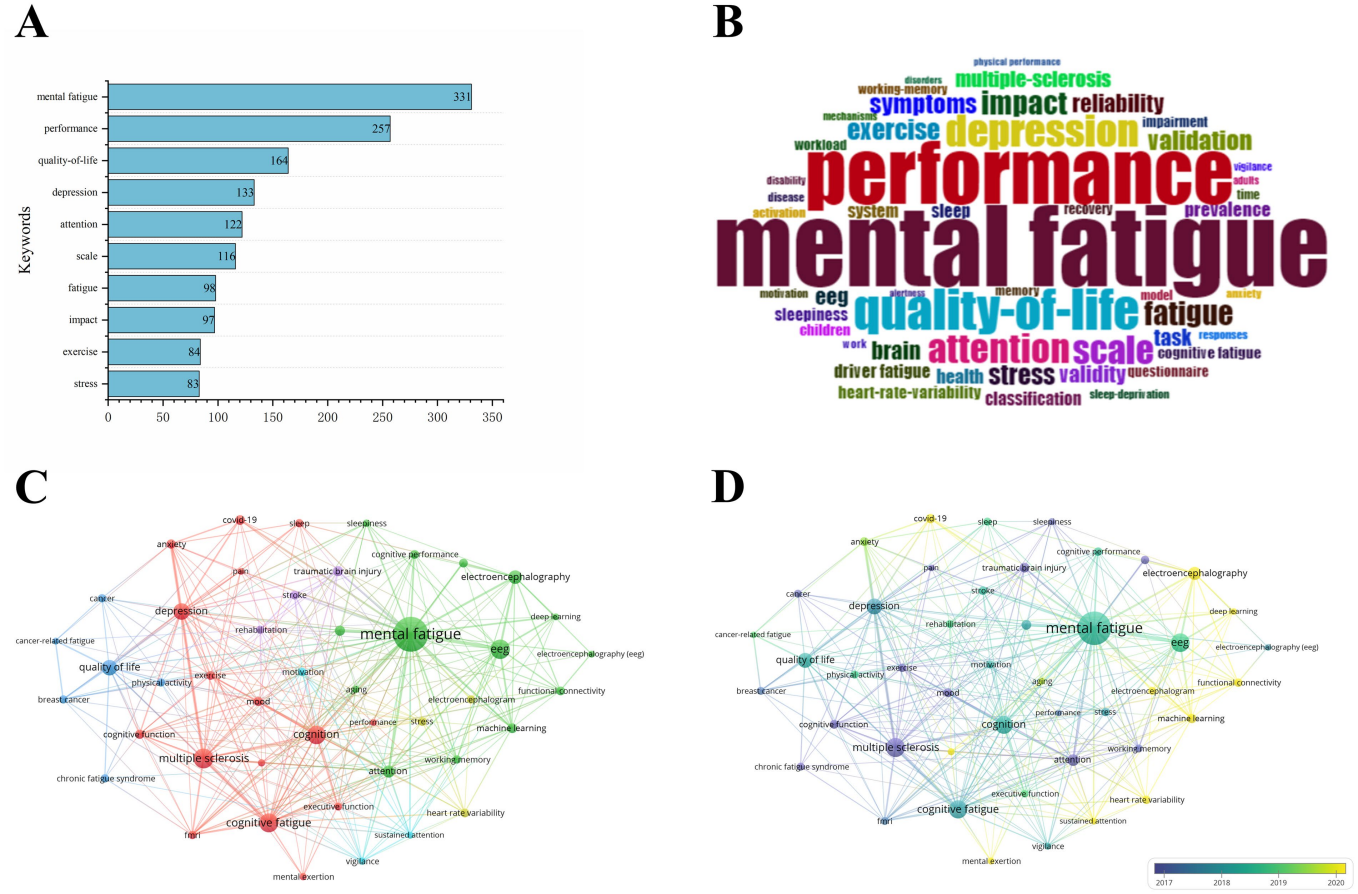
(A) Top 10 most frequent keywords. (B) Keywords Cloud map. (C) Network visualization map of keyword. (D) Overlay visualization map of keyword. Larger nodes mean higher frequency, and smaller nodes indicate lower frequency. A color represents a term in cluster analysis, and different colors represent different sub-clusters or classifications in this field. The distance between the nodes represents relevance, and nodes close to each other indicate high relevance. The thickness of connecting lines represents the strength of collaboration between the nodes.

Keywords mapping can reveal meaningful knowledge components and insights based on the patterns and strength of links between keywords of published papers. Identifying the most cited keywords can further inform our analysis of hotspots and the latest research trends. A total of 3,116 keywords were extracted, and the total frequency of occurrence was 10,566. The top 20 keywords about the objective assessment of cognitive fatigue were listed in Figure 6, and the top 10 keywords were visualized in Figure 6A. Figure 6B shows the Keywords Cloud, a visual representation of frequently used words. Keywords having higher density are presented in larger fonts and displayed in alphabetic order. Figure 6C shows the cluster analysis for keywords occurred at least 30 times, and four clusters were detected and the keywords with high frequency can accurately reveal the main topic. In cluster I with red color, the keywords with a high occurrence were mental fatigue, performance, attention, etc. In cluster II with green color, the keywords with a high occurrence were quality-of-life, depression, etc. In cluster III with blue color, the keywords with a high occurrence were exercise, sleep, reliability, recovery, etc. In cluster IV with yellow color, the keywords with a high occurrence were cognitive fatigue and multiple sclerosis. Figure 6D presents the overlay visualization map of keywords. A Keyword co-occurrence network is divided into 3 clusters of different colors according to the evolution of the research hotspots. The color of a keyword indicates the average publication time of articles containing that keyword.

**Figure 7:**
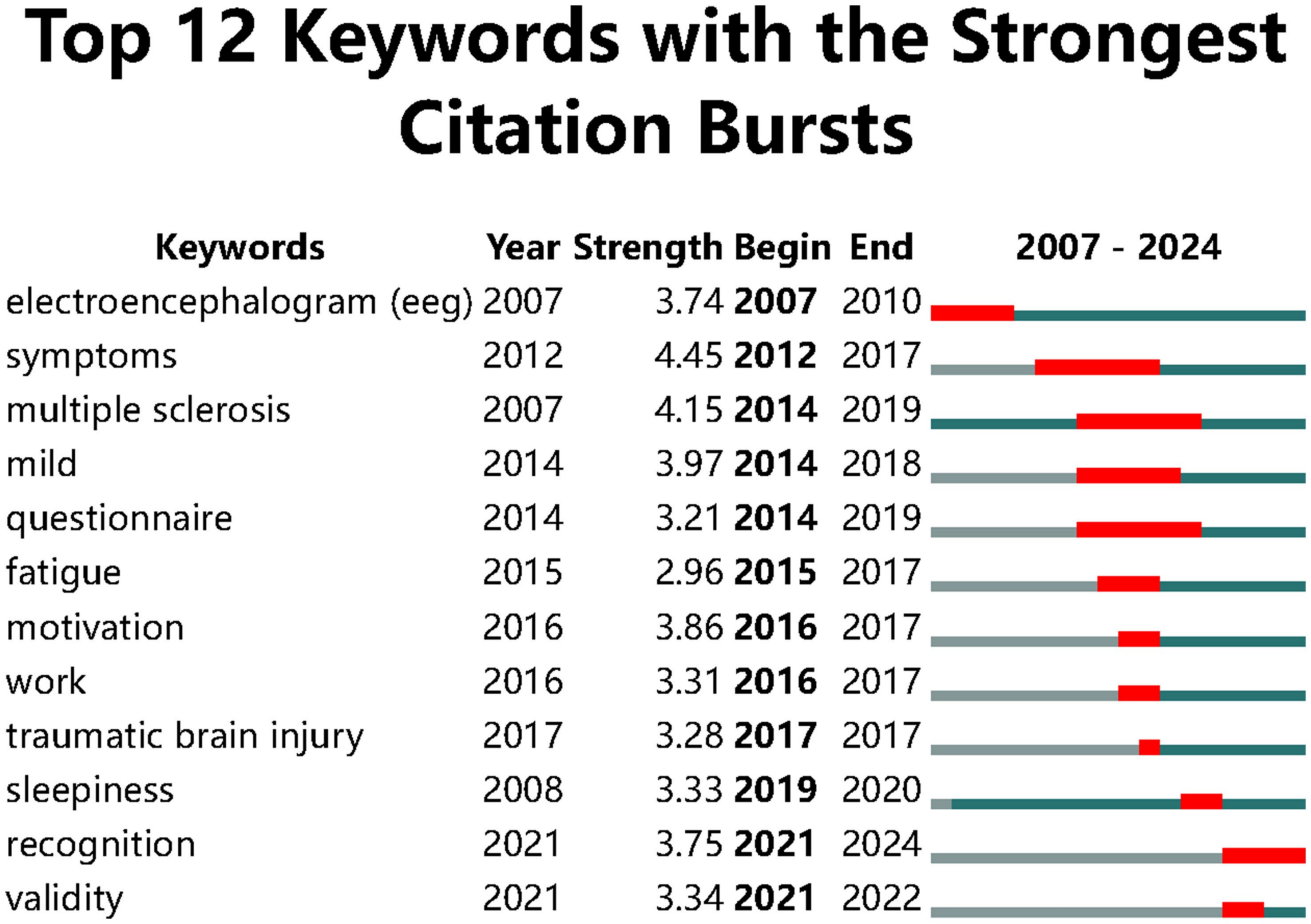
The top 12 keywords with the strongest citation burst.

We used CiteSpace to analyze keywords bursts to monitor the dynamic research changes. At present the top 207 keywords with the highest citation bursts were the indicators of frontier research topics. The early keywords (from 2007 to 2015) monitored with a burst were electroencephalogram (EEG) symptoms multiple sclerosis mild questionnaire and fatigue; but between 2016 and 2022 the keywords with the strongest citation bursts were motivation work traumatic brain injury sleepiness recognition and validity which is presented in Figure 7. This result indicates that cognitive fatigue caused by continuous work and traumatic brain injury has become a research development trend and more researchers focus on the rehabilitation of cognitive fatigue

### 3.6 Co-cited references and bursts detection

Co-cited references are those co-cited in the reference lists of other articles. The most co-cited references constitute hotspots and reflect the knowledge base and research trends. Co-cited references refer to documents that have been co-cited by the 300 studies included in the analysis. The top 10 co-cited references are listed in Table 6. The top one co-cited article entitled The Effects of Mental Fatigue on Physical Performance: A Systematic Review, is from Sports Medicine. This article summarized that duration and intensity of the physical task appear to be essential factors in decreasing the physical performance due to mental fatigue (Van Cutsem et al., 2017). All the co-cited references are related to sports, psychology, neuroscience, and engineering. Five references were co-cited more than 15 times. These articles summarized that objective analysis was applied in different domains for cognitive fatigue assessment. The most co-cited literature was published in Sports Medicine.

**Table 6:** Top 10 co-cited references.

Rank	Co-cited reference	Co-citation
1	Van Cutsem J, 2017, SPORTS MED, V47, P1569, DOI 10.1007/s40279-016-0672-0	34
2	Smith MR, 2019, J PSYCHOL, V153, P759, DOI 10.1080/00223980.2019.1611530	19
3	Smith MR, 2016, MED SCI SPORT EXER, V48, P267, DOI 10.1249/MSS.0000000000000762	19
4	Dimitrakopoulos GN, 2018, IEEE T NEUR SYS REH, V26, P740, DOI 10.1109/TNSRE.2018.2791936	16
5	Hu XY, 2020, J SAFETY RES, V72, P173, DOI 10.1016/j.jsr.2019.12.015	16
6	Smith MR, 2015, MED SCI SPORT EXER, V47, P1682, DOI 10.1249/MSS.0000000000000592	15
7	Gao ZK, 2019, IEEE T NEUR NET LEAR, V30, P2755, DOI 10.1109/TNNLS.2018.2886414	14
8	Russell S, 2019, EUR J SPORT SCI, V19, P1367, DOI 10.1080/17461391.2019.1618397	14
9	Qi P, 2019, ENGINEERING-PRC, V5, P276, DOI 10.1016/j.eng.2018.11.025	14
10	Martin K, 2018, SPORTS MED, V48, P2041, DOI 10.1007/s40279-018-0946-9	13

**Figure 8:**
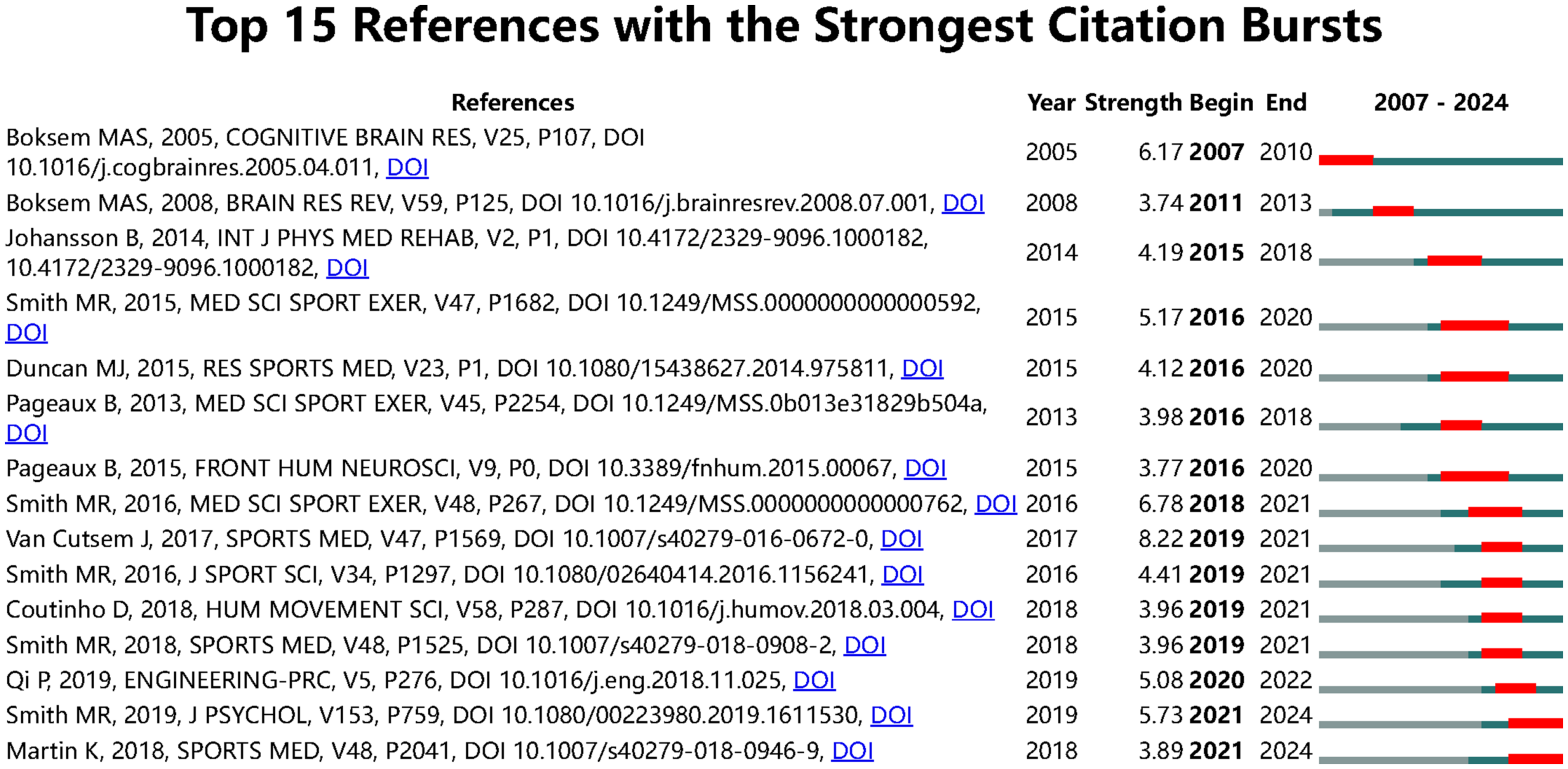
Top 15 references with the strongest citation bursts.

**Figure 9:**
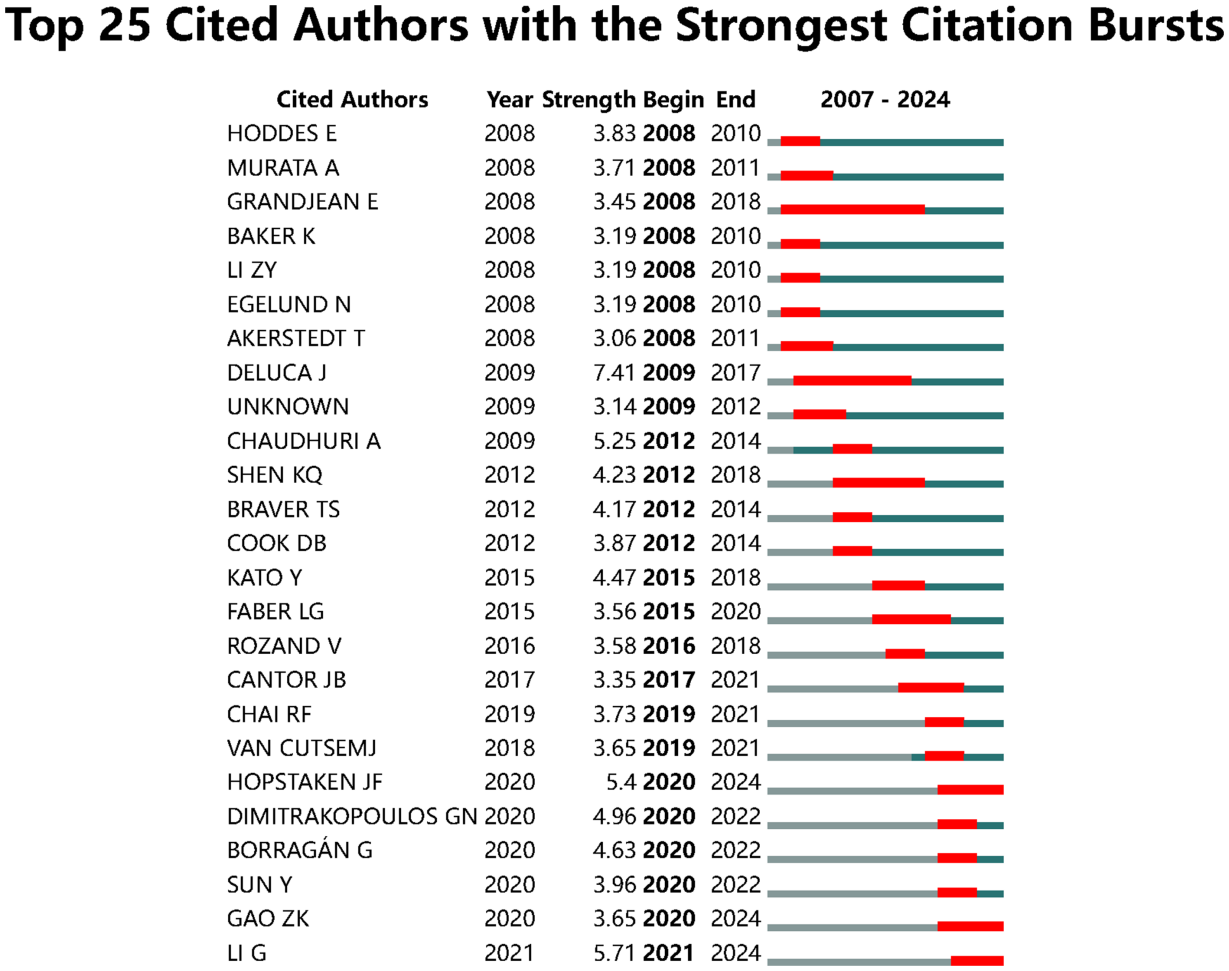
Top 25 co-cited authors with the strongest citation bursts.

**Figure 10:**
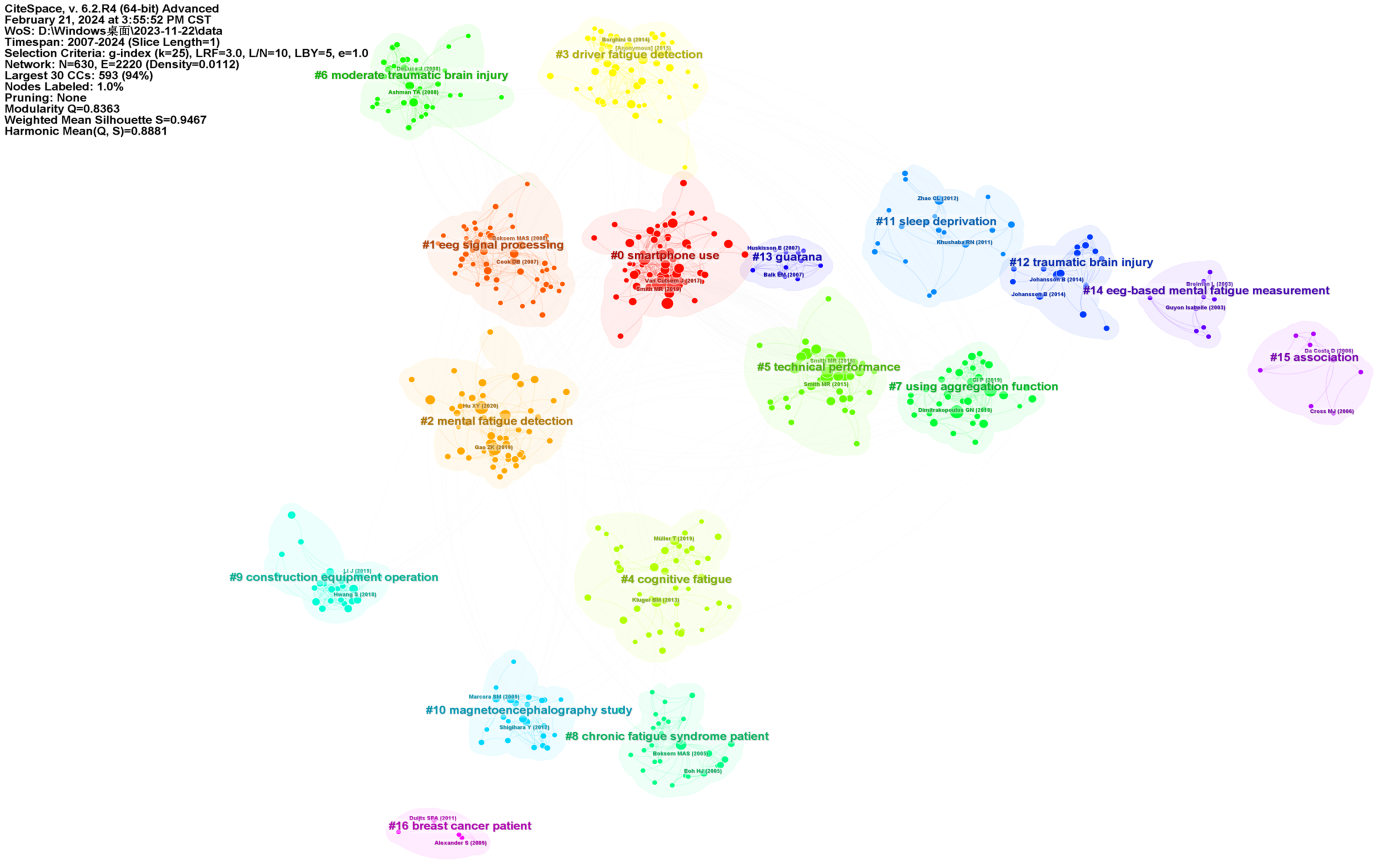
References co-citation network with correspondent clusters.

References with citation burst refer to documents that have been highly cited in a period. As shown in Figure 8 the threshold was set to top 15 in a one-year slice in CiteSpace; strong citation bursts with a minimal duration of 3 years were found in 15 co-cited references. The strongest burst was detected in a paper entitled Effects of mental fatigue on attention: An ERP study and its duration of high citation was 4 years (Mun et al., 2014). This paper focused on cognitive fatigue assessment through EEG event-related potential and spectral power analysis. Figure 9 shows that a strong citation burst with a minimal duration of 3 years was found in 25 co-cited authors. The dynamics of a field can be characterized in part by studies with citation bursts; these co-cited references and authors represented the current state of development for the objective assessment of cognitive fatigue. We also produced the reference co-cited network that included a total of 630 highly co-cited references (Figure 10). The network is organized by betweenness centrality, centrality scores are normalized to the unit interval of [0,1]. A node of high betweenness centrality is usually one that connects two or more large groups of nodes. A node with a strong betweenness centrality score has a great influence on a network. The *Q*-value indicating modularity was significant (*Q* = 0.8363), and the weighted mean silhouette score was highly credible (*S* = 0.9467). A total of 16 clusters with harmonic mean Q and S values above 0.8 were obtained.

## 4 Discussion

Bibliometric analysis is a tool to quantify the characteristics and scholarly impact of citation classics. In this study, the literature on the objective assessment of cognitive fatigue in the WOSCC database was analyzed using bibliometric methods based on R version 4.3.1, VOSviewer, and Citespace. This bibliometric study aimed to investigate the current status, hotspots, and trends in the objective assessment of cognitive fatigue. From the bibliometric analysis on the objective assessment of cognitive fatigue over the last 17 years, suggesting that more and more researchers are involved in the study of the cognitive fatigue detection (Figure 2).

Mapping the authors’ collaboration and countries/regions’ cooperation networks is an essential class of scientific social networks, which is used to show the key structure of scientific collaboration and the position of countries/regions. Traditionally, economic development will contribute to scientific and academic investment, thus developed countries may have a better impact on research productivity and quality. However, Table 2 shows the PRC has the largest number of publications in the field, which is also the only developing country in the top 10 countries, indicating that a large proportion of economics and spending was allocated to scientific research. However, the number of publications does not rank the same as the number of citations, indicating that the quality of articles from the USA is relatively better than those from the PRC. The reasons for this phenomenon may be the fact that developed countries established corresponding research earlier than developing countries, thus having better management of research papers. Network cluster analysis showed sufficient cooperation between countries and institutes. In addition, the PRC had the largest number of institutes. Surprisingly, this finding indicated that the PRC was the primary source of research publications in this field, instead of European or North American countries, which is different from the previous bibliometric study (Liu et al., 2018). Moreover, publications from the PRC in the field have begun to increase and are expected to contribute significantly to this field in the future.

In the cluster analysis, the top 10 authors have their own relatively fixed cooperative groups, namely only Bezerianos Anastasios and Sun Yu’s teams have cooperated (Figure 4). The red cluster represents a large group of authors, which indicates cooperation among these authors. Therefore, the distribution of scholars in this field is relatively scattered, so the cooperation of researchers needs to be strengthened on the cognitive fatigue detection. Although the number of published papers of all authors are not outstanding, the authors in these cluster still show good potential and prospects, as well as the diversity of author groups. It can be predicted that more high-yield authors will appear in these clusters in the future.

Journal analysis indicated the quality and influence of these journals and showed the leading position of the USA in this research field. In order to gain insight into the research related to the application of objective assessment of cognitive fatigue, we can select publications in these journals to provide references for future research directions. The IF of these journals can also reflect the importance and priority of the field. Besides, the dual-map overlay of journals also indicated that the research on cognitive fatigue is also in line with the essence of researching cognition of human (Figure 5).

In co-cited references analysis, most researchers obtained citations from a few main journals in their respective fields of expertise. When the researchers deviated from these core journals, their citation frequency and impact were weakened. Consequently, this tendency led to a large percentage of citations stemming from a few core journals.

**Figure 11:**
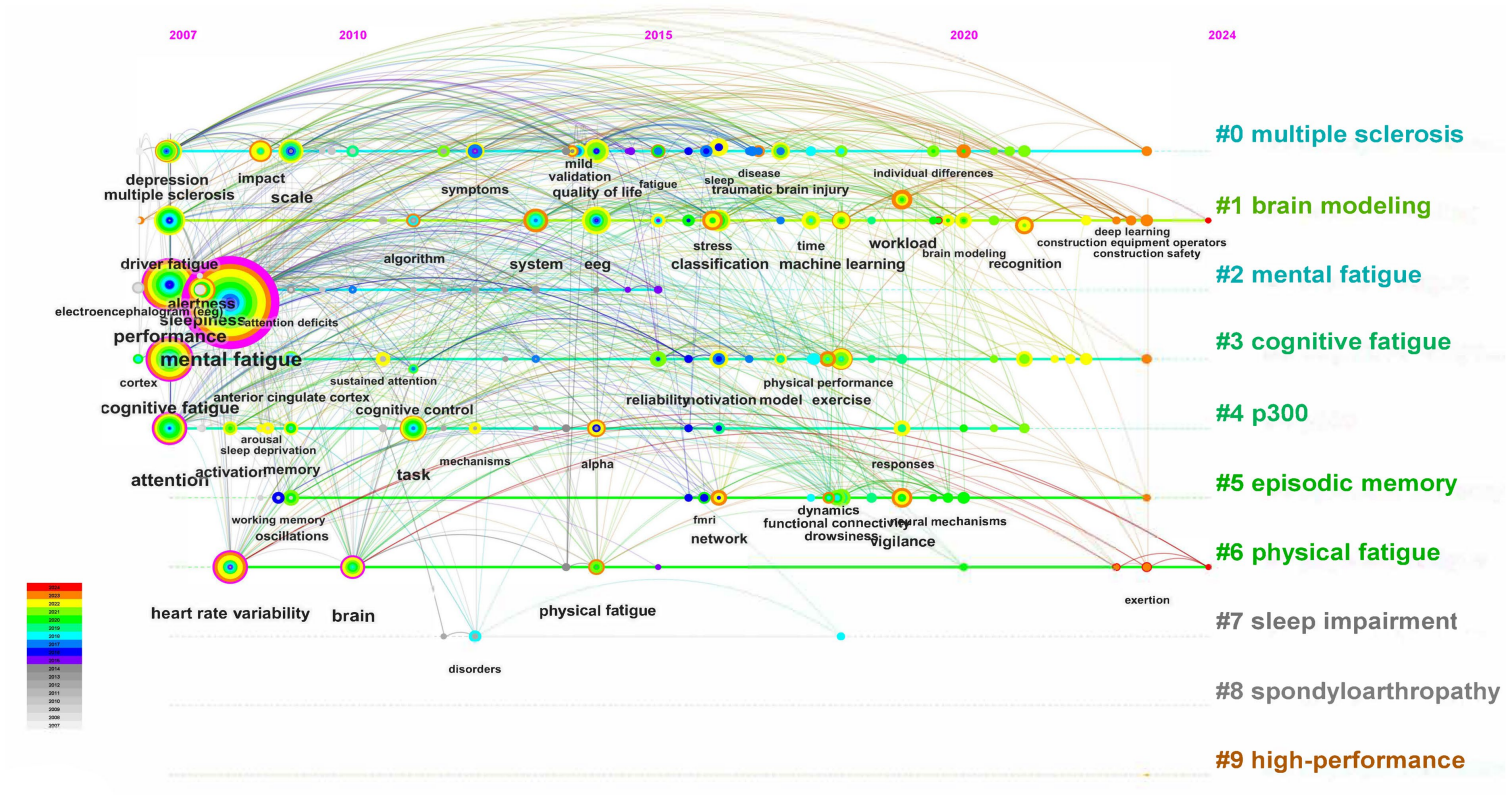
The timeline map of keywords. Keywords in this figure are represented by nodes, and the colors show the average year of publication for each node. The Burstiness of keyword co-occurrence is proportional to each cross. The co-occurrence network is weighted on total link strength across different keyword nodes and scored on the average publication years. Red tree-rings growth from the center of nodes and indicate an important burst of keywords, while a peripheral purple tree-rings indicate an ancient burst in citations.

**Figure 12:**
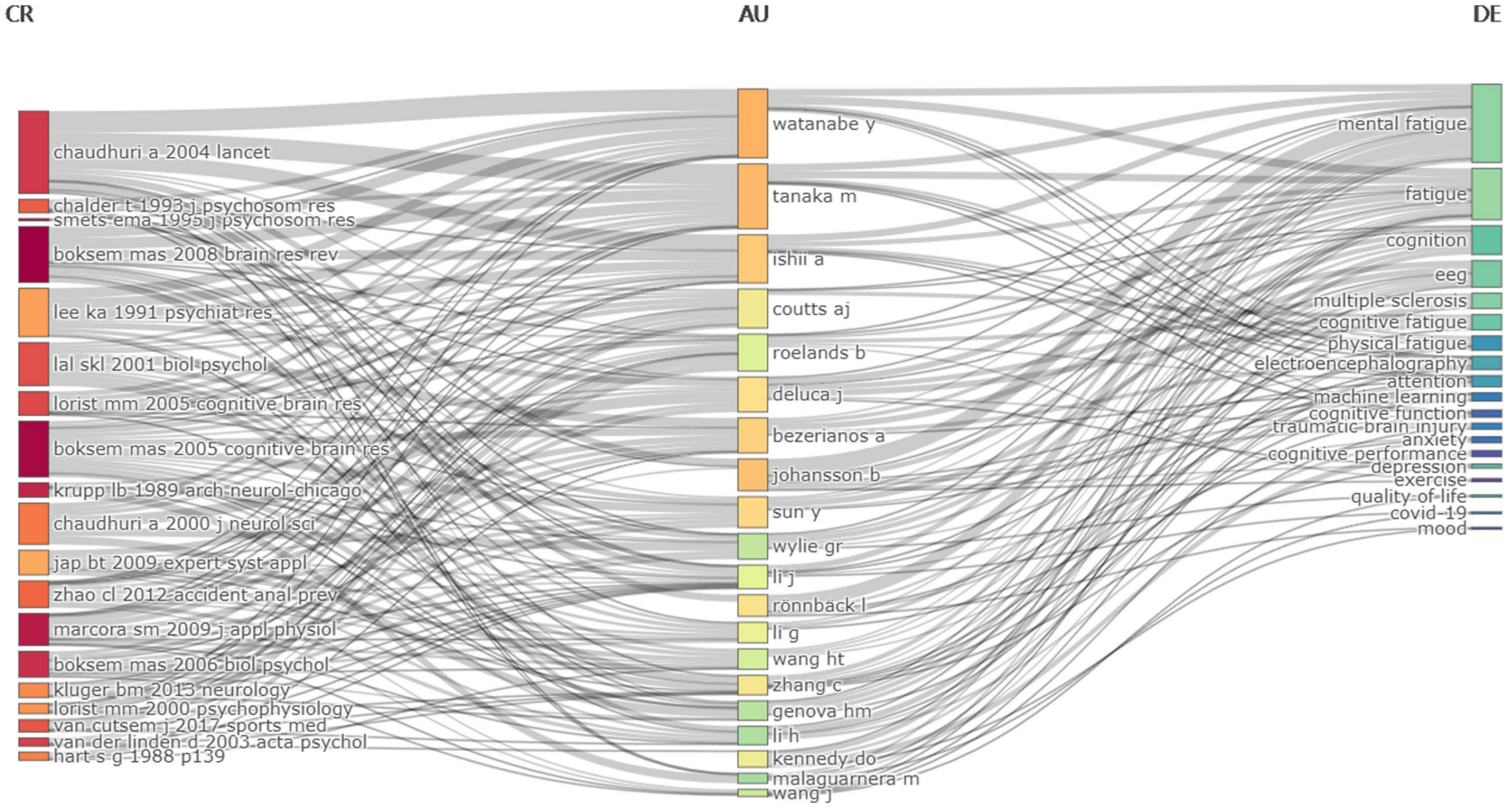
Three-field plot of the Keywords Plus analysis (middle field: authors; left field: most cited references; right field: keywords plus).

A map of keywords can reflect hot topics of research. In addition, keywords can represent the core point of an article. Keywords analysis can be used for detecting research topics, as well as for monitoring the research frontier transitions of a specific knowledge domain. Interestingly, the most frequent keyword was mental fatigue rather than cognitive fatigue, the same result was also shown in keyword network clustering analysis. Indeed, most of the literature suggests that cognitive, mental, and brain fatigue refer to the same meaning. However, more authors considered that “mental fatigue” is more appropriate as it includes emotion and motivation rather than just cognition (Marcora et al., 2009; Brownsberger et al., 2013; Pageaux et al., 2015; Smith et al., 2015; Bijleveld, 2023; Sengupta et al., 2017; Sievertsen et al., 2016). Surprisingly, timeline visualization of keywords network has shown that the term “cognitive fatigue” has occurred more frequently in recent years, implying that more researchers are concentrating on the effect of high-workload tasks on one’s cognition (Figure 11). Furthermore, this figure indicates that P300, an EEG component extracted by synchronous averaging over time locked single-trial of event-related potential, is also a hotspot for research in the application of objective assessment of cognitive fatigue (Sabeti and Boostani, 2017; Lamti et al., 2019). Besides, overlay visualization map of keywords also indicates that cognitive fatigue assessment based on neurophysiology and artificial intelligence has become popular in recent years (Figure 5). The three-field plot of the Keywords Plus analysis in Figure 12 also shows the relationships between institutes, authors, and keywords.

Keywords cluster analysis also found the enthusiasm of researchers to use objective analysis in driver, surgeons and pilots fatigue detection, which found EEG, fNIRS, HRV, electrooculogram (EOG) and eye-tracking measures can deal with a variety of challenges (Whelehan et al., 2021; Stancin et al., 2021; Gao et al., 2021; Figure 6C). EEG-based frequency-domain (FD) (Chen et al., 2022b), brain connectivity (BC) (Chen et al., 2022b; Wang et al., 2020; Wang et al., 2021a; Xu T. et al., 2022) and fusion entropy (Wang et al., 2019) have been proven to be reliable indicators for estimating driving and pilots fatigue state (Wang et al., 2022). In general, most objective measures can be classified either as neurophysiological, physiological, or behavioral. Neurophysiological measures include EEG and fNIRS (Leanne et al., 2009). Physiological measures include electrocardiography (Veltman et al., 1996) and eye tracking (pupil dilation (Pomplun and Sunkara, 2003), blink frequency and blink duration (Tsai et al., 2007), and saccades (Ahlstrom and Friedman-Berg, 2006). Behavioral measures include keystroke dynamics, mouse tracking, and body positioning (Mota and Picard, 2003). However, the processes for traditional feature extraction of these indicators are time-consuming, and the intrinsic relations between channels may be neglected (Wang et al., 2021b). Moreover, the accuracy, robustness, temporal resolution, and latency of these approaches are suboptimal and, in certain contexts, do not respond to changes in task load at all (Rozado and Dunser, 2015; Wilson, 2002). This is due to the measurement noise, artifacts induced by a subject’s movements, and the limited amount of training data available. Developing a robust and accurate metric that can quickly and efficiently reflect the cognitive fatigue states remains a challenging task.

In recent years novel strategies have been explored to overcome these limitations. Plenty of innovative deep neural networks have been developed for driver and pilots fatigue detection

such as attention-based multiscale convolutional neural network-dynamical graph convolutional network (AMCNN-DGCN) (Wang et al., 2021b) approximate empirical kernel map-fusion-based support vector machine (AEKM-Fusion-SVM) (Chen et al., 2022b) and Self-Attentive Channel-Connectivity Capsule Network (SACC-CapsNet) (Chen et al., 2023). Another approach is multimodal data fusion a group of techniques that allow the incorporation of data from different sources (from the same or different modalities) to infer meaningful information that is inaccessible by using a single independent source (Liggins et al., 2009; Majumder et al., 2018). The concept of data fusion from different sources is not new. Indeed humans and animals use multiple senses (e.g., vision smell and hearing) to obtain valuable information and to be able to assess different situations for survival. For instance in situations with low visibility such as in darkness or dense fog the senses of touch and hearing can be used to overcome visual limitations. Therefore combining different senses helps construct a more accurate assessment of a given situation. Similarly, robust and enhanced results can be achieved by utilizing multiple measures together to assess cognitive fatigue (Chanel et al., 2006; Wilson and Russell, 2007; Maarten Andreas et al., 2014; Anne-Marie et al., 2017) such as multimodal data fusion of FD and BC based on EEG (Chen et al., 2022b) EEG and EOG (Wang et al., 2019) EEG and eye tracking (Lian et al., 2024) as well as convolutional neural network and attention (CNN-Attention) structure (Xu et al., 2023). These methods can effectively improve the fusion efficiency of the features from different dimensions we believed that new technological and conceptual developments will fuel the application of approaches for cognitive fatigue detection

This study has some limitations: (1) the data were retrieved from the single database of the WOSCC, did not include other databases, excluded non-English papers, and there may be a certain bias in the sources of the included documents. However, most existing databases of biomedical and life science, such as PubMed or Embase, do not propose full text and citation analyses, which are mandatory for bibliometric analysis. (2) Part of the results were standardized manually before analysis to reduce the deviation caused by different expressions of the same concept, and such errors can only be reduced rather than eliminated. Notably, citations are influenced by many factors, including random circumstances, personal relationships and open access status. For example, novel papers may suffer from delayed recognition and need a sufficiently long citation time window before reaching the status of a large hit. (3) Only articles are included, which will miss some representative publications, but this is to ensure the formality and completeness of the included literature. (4) The total number of pieces of literature included is small.

## 5 Conclusion

In this study we analyzed the publication information related to the application of objective assessment of cognitive fatigue from 2007 to 2023, including country, institute, author, publication journal, keyword, and co-cite reference. Facing the exponential number of publications, bibliometric analyses can identify the knowledge domains and emerging trends of a specific field of research. Cooperation between the author and the organization embodies good communication and cooperation. Future research on cognitive fatigue assessment will still focus on EEG, fNIRS, HRV, EOG and eye-tracking measures, especially on developing innovative deep neural networks and the multimodal fusion of different modalities. This study may be useful for determining future research topics related to cognitive fatigue, and helpful for research of timely identifying the states of cognitive fatigue and intervention. The research hotspots based on these publications were analyzed comprehensively, it could be predicted from bibliometric analysis that the trends such driver and pilots fatigue detection could be the hotspots and frontiers of research in the future.
